# Increased HLA-G Expression in Term Placenta of Women with a History of Recurrent Miscarriage Despite Their Genetic Predisposition to Decreased HLA-G Levels

**DOI:** 10.3390/ijms20030625

**Published:** 2019-02-01

**Authors:** Moniek H. C. Craenmehr, Iris Nederlof, Milo Cao, Jos J. M. Drabbels, Marijke J. Spruyt-Gerritse, Jacqueline D. H. Anholts, Hanneke M. Kapsenberg, Janine A. Stegehuis, Carin van der Keur, Esther Fasse, Geert W. Haasnoot, Marie-Louise P. van der Hoorn, Frans H. J. Claas, Sebastiaan Heidt, Michael Eikmans

**Affiliations:** 1 Department of Immunohematology and Blood Transfusion, Leiden University Medical Center, Albinusdreef 2, 2333 ZA Leiden, The Netherlands; m.h.c.craenmehr@lumc.nl (M.H.C.C.); iris.nederlof@xs4all.nl (I.N.); milocao@outlook.com (M.C.); J.J.M.Drabbels@lumc.nl (J.J.M.D.); M.J.Spruyt-Gerritse@lumc.nl (M.J.S.-G.); J.D.H.Anholts@lumc.nl (J.D.H.A.); J.M.Kapsenberg@lumc.nl (H.M.K.); J.A.Stegehuis-Kamp@lumc.nl (J.A.S.); C.van_der_Keur@lumc.nl (C.v.d.K.); G.W.Haasnoot@lumc.nl (G.W.H.); F.H.J.Claas@lumc.nl (F.H.J.C.); S.Heidt@lumc.nl (S.H.); 2 Department of Laboratory Medicine, Laboratory of Medical Immunology, Radboud University Medical Center, Geert Grooteplein Zuid 10, 6525 GA Nijmegen, The Netherlands; Esther.Fasse@radboudumc.nl; 3 Department of Obstetrics and Gynaecology, Leiden University Medical Center, Albinusdreef 2, 2333 ZA Leiden, The Netherlands; M.L.P.van_der_Hoorn@lumc.nl

**Keywords:** HLA-G, immunohistochemistry, placenta, pregnancy, recurrent miscarriage

## Abstract

Human leukocyte antigen (HLA)-G is an immune modulating molecule that is present on fetal extravillous trophoblasts at the fetal-maternal interface. Single nucleotide polymorphisms (SNPs) in the 3 prime untranslated region (3′UTR) of the *HLA-G* gene can affect the level of HLA-G expression, which may be altered in women with recurrent miscarriages (RM). This case-control study included 23 women with a medical history of three or more consecutive miscarriages who delivered a child after uncomplicated pregnancy, and 46 controls with uncomplicated pregnancy. Genomic DNA was isolated to sequence the 3′UTR of *HLA-G*. Tissue from term placentas was processed to quantify the HLA-G protein and mRNA levels. The women with a history of RM had a lower frequency of the *HLA-G* 3′UTR 14-bp del/del genotype as compared to controls (Odds ratio (OR) 0.28; *p* = 0.039), which has previously been related to higher soluble HLA-G levels. Yet, HLA-G protein (OR 6.67; *p* = 0.006) and mRNA (OR 6.33; *p* = 0.010) expression was increased in term placentas of women with a history of RM as compared to controls. In conclusion, during a successful pregnancy, HLA-G expression is elevated in term placentas from women with a history of RM as compared to controls, despite a genetic predisposition that is associated with decreased HLA-G levels. These findings suggest that HLA-G upregulation could be a compensatory mechanism in the occurrence of RM to achieve an ongoing pregnancy.

## 1. Introduction

About 1% to 2% of couples trying to conceive experience recurrent miscarriages (RM) [1]. Accepted etiological categories for RM include chromosomal abnormalities, uterine anatomic abnormalities, and antiphospholipid antibody syndrome. However, a significant proportion of the couples trying to conceive do not know the underlying cause for this recurring problem [2], leaving them with a burden of uncertainty.

During pregnancy, the maternal immune system needs to accept the semi-allogeneic fetal tissue. For this reason, several mechanisms are at play at the fetal-maternal interface. The absence of the human leukocyte antigen (HLA) class I antigens A and B and HLA class II on fetal trophoblast cells helps to prevent allorecognition by T and B cells, whereas the presence of HLA-C, HLA-E, HLA-F, and HLA-G provide self-signals to control (natural killer) Natural Killer (NK) responses [3,4]. Low levels of HLA-G have been associated with RM [5,6]. By alternative splicing, the HLA-G pre-mRNA can give rise to seven different isoforms, of which four are membrane-bound (HLA-G1, -G2, -G3, and -G4) and three are soluble (HLA-G5, -G6, and -G7) [7]. Whereas, in healthy tissue, membrane-bound HLA-G is only expressed on trophoblasts, the soluble form of HLA-G can be detected in various body fluids, such as amniotic fluid, blood, and seminal plasma [8,9,10]. One mechanism leading to the generation of soluble (s)HLA-G1 is the cleavage of membrane-bound HLA-G from the cell surface by the activity of metalloproteinases [11].

Several polymorphisms are present in the 3 prime untranslated region (3′UTR) of the *HLA-G* gene. Since the 3′UTR is targeted by microRNAs (miRNA) that can negatively influence expression, polymorphisms in this region may have an influence on the efficiency of miRNA binding and, consequently, on the level of HLA-G expression and on pregnancy outcome. The 14 bp insertion/deletion polymorphism affects the stability of HLA-G mRNA and thereby the expression of HLA-G [12]: the insertion is associated with low levels of sHLA-G [13]. The presence of the +3187A allele is associated with decreased mRNA stability and decreased HLA-G expression [14]. The presence of a guanine at position +3142 increases the affinity of miR-148a, miR-148b, and miR-152, which leads to the downregulation of HLA-G expression [15].

We analyzed the *HLA-G* 3′UTR genotype of women with a history of RM and of a control group of women with no history of RM. We also analyzed the *HLA-G* 3′UTR genotype of their offspring. The combination of multiple polymorphic sites was used to generate 3′UTR haplotypes. In addition, we studied HLA-G mRNA and protein expression levels in term placentas of women with successful pregnancies in both study groups.

## 2. Results

### 2.1. Patient Characteristics

Characteristics of the RM group and control group are listed in Table 1. The groups did not differ in maternal age and gestational age (GA) at delivery. As expected, the women in the RM group had fewer previous live born children when compared to the control women (*p* < 0.001). Of the RM group, 65.2% had no children, as compared to 19.6% in the control group.

### 2.2. HLA-G Polymorphisms and Haplotypes

We analyzed multiple SNPs to distinguish eight haplotypes of the 3′UTR in exon 8 of *HLA-G*. All of the genotyped SNPs fit the Hardy–Weinberg expected proportions in both groups of women and in their offspring (Tables S1 and S2). No differences in frequency for individual SNPs or in haplotype distribution was found between groups (Tables S3 and S4), except for the 14-bp indel polymorphism. We found a higher frequency of *HLA-G* 14-bp ins/del heterozygotes in RM women (65.2%) as compared to control women (36.4%) (OR 3.28; *p* = 0.026) and a lower del/del genotype (17.4% compared to 43.2%) (OR 0.28; *p* = 0.039), whereas the frequencies of the ins/ins genotype are very similar in both groups (17.4% vs. 20.5%) (Table 2). Nevertheless, the allelic frequencies of deletion and insertion do not differ significantly between RM and controls (Table 2). The 14-bp insertion is known to influence mRNA stability [16], resulting in lower HLA-G expression [13]. The children in both groups did not differ in the frequency of individual SNPs (Table S6), haplotypes (Table S5) and 14-bp indel (Table 3).

### 2.3. Placental HLA-G Expression Is Elevated in Women with History of RM

**Figure 1:**
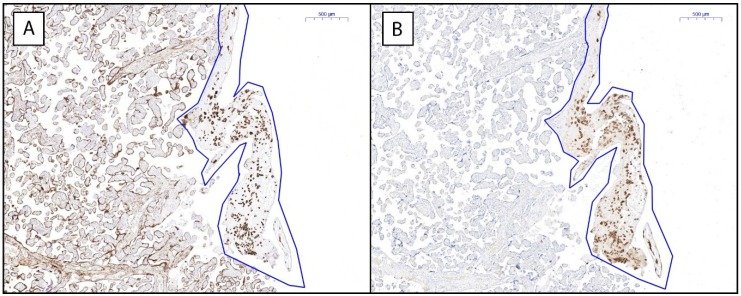
Expression of trophoblast cell marker and HLA-G in term placenta. Representative examples of staining for (**A**) trophoblasts with cytokeratin marker anti-cytokeratin antibody (CAM5.2) and (**B**) all HLA-G isoforms with marker MEM-G2. Decidual parts of the placenta were annotated to specify the area for analysis.

Trophoblasts were stained in the term placentas by means of immunohistochemistry with an anti-cytokeratin antibody (CAM5.2) (Figure 1A). On the sequential slides, an antibody recognizing the free heavy chain of all HLA-G isoforms (MEM-G2) was applied (Figure 1B). Expression of HLA-G was confined to the cytokeratin positive cells in the decidua basalis, as determined by double staining experiments by immunofluorescence (Figure S2). We annotated the decidual part of the placental tissues and quantified the extent of staining within these annotations. The Spearman’s correlation coefficient of the inter-observer reproducibility for our approach of quantitation was *r* = 0.79. In the Bland–Altman plot of inter-observer measurements (Figure S3), most of the values ranged within a mean ± two SD, meaning that the reproducibility of the measurement is acceptable [17].

**Figure 2:**
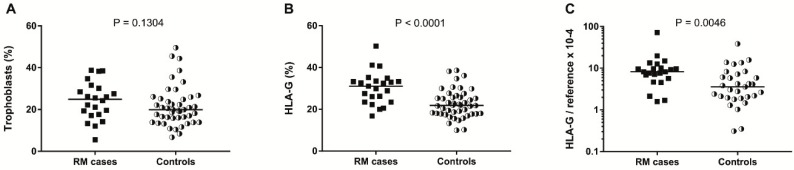
(**A**) Percentage positivity for trophoblast staining. No difference was observed in trophoblast staining between women with a history of RM and controls. (**B**) Percentage positivity for HLA-G staining. A higher HLA-G protein expression was observed in the decidual part of the placenta of women with a history of RM compared to controls. (**C**) HLA-G mRNA expression was measured in the placentas of women with a history of RM and controls. HLA-G mRNA expression was increased in term placenta of women with a history of RM as compared to controls.

No significant difference was observed in the extent of trophoblast staining between groups (Figure 2A). However, the extent of decidual HLA-G protein expression was elevated in the placentas of women with a history of RM (median 32.6%) as compared to the control group (median 21.9%, *p* < 0.0001) (Figure 2B). The HLA-G expression was similar in placentas of women who gave birth to their firstborn as compared to women who already had a successful previous pregnancy (Figure S4). Using the median expression in the controls, the RM subjects were divided into either low or high HLA-G protein expression groups (Table 4). From RM cases, 87.0% belonged to the high HLA-G protein expression group (OR 6.67, 95% CI: 1.74–25.57; *p* = 0.006).

To verify the differences that were observed at the protein level, we analyzed the mRNA expression of HLA-G in homogenates of term placentas from both groups. For this, we developed primers targeting exon 2 and 3 of the *HLA-G* gene, so all HLA-G isoforms were recognized. To verify that the primers only recognize HLA-G, and not HLA-C, their specificity was checked by sequencing of the amplicons. The mean cycle quantification (Cq) value for all placentas was 25.2 ± 2.0 (range 21–33), indicating expression that was well above background. Similar to what was observed for HLA-G protein expression, the placentas of women with a history of RM had a 2.3-fold higher HLA-G mRNA expression than women without a history of RM (median relative level 8.2 versus 3.6, *p* < 0.005) (Figure 2C). RM subjects were divided into either low or high HLA-G mRNA expression groups (Table 5). From RM cases, 86.4% belonged to the high HLA-G mRNA expression group (OR 6.33, 95% CI: 1.56–25.71; *p* = 0.010). No correlation was found between the maternal and fetal *HLA-G* genotype with HLA-G expression.

We wondered whether the higher placental HLA-G expression in the RM group was accompanied by a lower level of miRNAs. Members of the miR-148 family and miR152 and miR-365 have been identified to target the 3′UTR of *HLA-G* [18,19]. Cq values for miR-148a, miR148b, and miR-152 ranged between 16 and 29. The Cq values for miR-365 ranged between 25 and 39. After normalization for two reference miRNAs, no difference was observed between groups in the levels of miR-152 and miR-365 (Figure S5). Placental miR-148a (*p* = 0.0009) and miR-148b levels (*p* = 0.0154) were higher in the RM group as compared to controls. Thus, increased HLA-G expression in the RM group was not accompanied by decreased miRNA levels.

### 2.4. HLA-G Expression in First Trimester Miscarriage Material and Elective Abortions Is Similar

**Figure 3:**
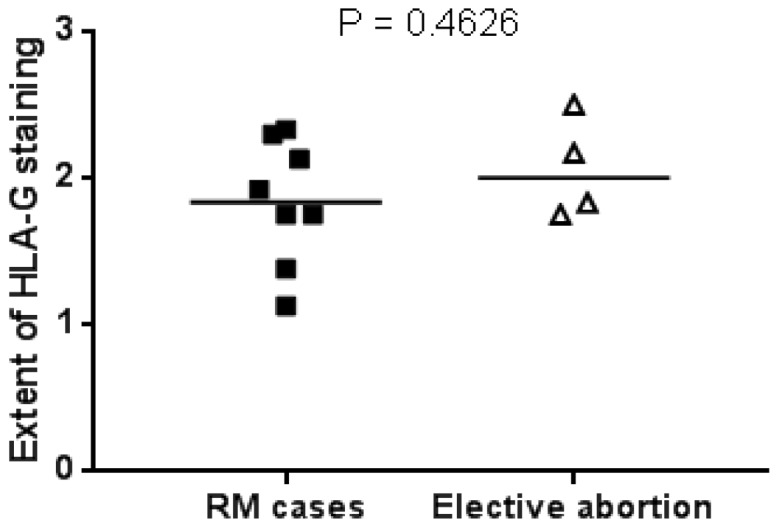
Amount of HLA-G staining in first trimester miscarriage and elective abortion material. HLA-G positive parts in the extravillous trophoblast (EVT) regions of the placental slides were scored to be (1) minimally, (2) moderately, or (3) intensely stained.

Since HLA-G protein expression was elevated in term placentas after successful pregnancies, we additionally analyzed HLA-G expression in first trimester placentas, using the same antibody for immunohistochemistry. To this aim, we collected first trimester miscarriage of patients with a history of RM and elective abortion material and stained slides for HLA-G. Since the decidua could not be clearly defined in this early material, the HLA-G positive region was selected to semi-quantitatively score the extent of protein staining. The average score for each group is shown in Figure 3. No difference in HLA-G protein expression was found between early miscarriages and elective abortions.

## 3. Discussion

In this study, we investigated the *HLA-G* genotype and HLA-G mRNA and protein expression in term placentas of women with a history of RM and of women with no such history. A homogenous well-defined case group of women with at least three consecutive unexplained RM within 20 weeks of gestation was included. A lower frequency of the *HLA-G* 3′UTR 14-bp deletion genotype was observed in the case group, suggesting that genetic predisposition to a low level of HLA-G played a role in the etiology of previous RM. In the current successful pregnancies, a significantly higher HLA-G protein and mRNA expression was found in the placenta of the RM group when compared to the control group.

The most studied polymorphism in exon 8 of 3′UTR of the *HLA-G* gene is the 14 bp indel polymorphism, which has been associated with altered HLA-G expression. The insertion genotype is associated with low levels of sHLA-G [13]. In addition, the fetal 14-bp ins/ins genotype has been associated with lower surface expression of HLA-G on first trimester trophoblast cells than the 14-bp del/del genotype [20]. We did not find any differences in fetal *HLA-G* 3′UTR haplotypes or individual SNPs between both groups. However, we found a higher frequency of *HLA-G* 14-bp ins/del heterozygotes in RM women (65.2%) as compared with control women (39.1%), and a lower frequency of *HLA-G* 14 bp del/del homozygotes (17.4% and 43.2%, respectively). This is consistent with some studies [21,22,23], but not others [24,25]. Since several studies have focused on the *HLA-G* 14-bp polymorphism in RM with controversial or inconclusive results, Wang et al. performed a meta-analysis [26], which suggested that the *HLA-G* 14-bp insertion allele was associated with an increased risk of RM. In 2014, yet another meta-analysis indicated that there was only an association between the *HLA-G* 14-bp indel polymorphism and RM in patients with three or more miscarriages [27]. In the present study, we have not addressed polymorphisms in the *HLA-G* promotor region, but they may be associated to RM, as recently shown [28].

Both the individual SNPs and the most common extended 3′UTR haplotypes of *HLA-G* were studied in the group of women with a history of RM and controls. *HLA-G* haplotype distribution and the frequencies of individual SNPs in the 3′UTR region of *HLA-G* were neither significantly different between the groups of women, nor in their offspring (Tables S3–S6). Studies have shown that individual SNPs in the 3′UTR region of *HLA-G* are not significantly associated with RM, but that the UTR-4 haplotype seemed to be protective against RM [29,30]. Similarly, we observed a lower incidence of the *HLA-G* UTR-4 haplotype in women with RM (10.9% in RM women vs. 15.9% in control women) (Table S3). Remarkably, the *HLA-G* UTR-4 haplotype was more frequently present in the offspring of women with RM than in the offspring of controls (21.7% vs. 13.3%, respectively) (Table S5) and less frequently in miscarriage material from women with RM (10%). Even though these results were not statistically significant, which is possibly due to limited sample size, collectively they support the idea that this haplotype might have a protective effect in uncomplicated pregnancy.

HLA-G in the placenta is suggested to play a role in the induction of immunological tolerance at the fetal-maternal interface, by functioning as a trophoblast-restricted inhibitory ligand of maternal immune cells. Only a few studies have focused on HLA-G protein expression in the placentas of women with a history of RM, with contradicting results [31,32]. Remarkably, the present immune-histochemical analysis of term placentas of successful pregnancies showed a significantly higher HLA-G protein expression in women with a history of RM compared to controls, although this RM group had a lower frequency of the 14 bp del/del genotype. This is not in line with results previously found in peripheral blood [21,33] and suggests that local regulation is involved. HLA-G was mostly confined to the trophoblast areas at the fetal-maternal interface (decidua basalis), as determined by double label immunofluorescence experiments (Figure S2), and the level of HLA-G expression was independent of previous pregnancies.

Since the level of HLA-G expression can depend on the differentiation status of extravillous trophoblasts (EVTs), as determined by in vitro studies using isolated primary trophoblasts [34], it is unclear whether the observed differences in HLA-G expression are a direct consequence of transcriptional regulation or a secondary consequence of an altered differentiation status of the EVTs. Possibly, for a successful pregnancy to occur after previous RM, a compensatory mechanism resulting in high HLA-G protein expression is in place. When comparing first trimester miscarriage material of women with a history of RM and the material of elective abortions, we did not observe a difference in HLA-G expression between both groups, suggesting that successful pregnancy in women with a history of RM is due to high fetal HLA-G expression in the current pregnancy. Besides HLA-G, other molecules and immune interactions may be involved in the immune-regulation, leading to successful pregnancy.

We found higher miR-148a and miR-148b expression in the term placentas of the RM group despite the elevated HLA-G expression. Apparently, either these miRNAs do not bind or binding does not result in post-transcriptional repression of HLA-G. This leads us to hypothesize that the higher HLA-G protein expression in the RM group may be the result of an epigenetically-regulated compensatory mechanism to achieve an ongoing pregnancy in patients with a history of RM. Alternatively, the higher HLA-G protein expression in the case group may be an epiphenomenon resulting from previous miscarriages. It is possible that the elevated HLA-G in the term placentas of women with RM is the result of proteolytic cleavage of the membrane bound HLA-G1 isoform resulting from activity of metalloproteases, leading to elevated sHLA-G levels. The antibody recognizing MEM-G2 in our immune-histochemical assays does not distinguish membrane bound HLA-G from soluble HLA-G. Previous miscarriages could lead to increased metalloprotease (MMP) levels [35], which in turn lead to increased proteolytic shedding of HLA-G1 [11]. MMP2 and MMP9 mRNA expression was not elevated in the term placentas of women with a history of RM as compared to controls (Figure S6), but this does not fully exclude the involvement of MMPs, since their activity was not tested in the current setting.

In conclusion, whereas women with RM have a genetic predisposition to lower HLA-G levels, HLA-G expression is increased in the placenta of ongoing pregnancies after RM. This implies that HLA-G upregulation could be a compensatory mechanism in the occurrence of RM to achieve an ongoing pregnancy. Whether the higher HLA-G expression in the ongoing pregnancy after RM is a cause or a consequence of the successful pregnancy remains to be established. Future studies should be concentrated on further establishing the role of HLA-G in complicated pregnancies. Measurement of maternal sHLA-G may provide further insight on the prognosis of the outcome of pregnancies in women with a history of RM.

## 4. Materials and Methods

### 4.1. Subjects and Materials

This case control study included women with a medical history of RM who delivered a child after uncomplicated pregnancy. These women visited the Department of Obstetrics and Gynaecology, Leiden University Medical Center (LUMC) between 2012 and 2015, and no underlying cause for RM was found after a full clinical workup according to the local guidelines, which are in line with the international ESHRE guidelines. Twenty-three women with a history of at least three miscarriages and an uncomplicated singleton pregnancy were included in this study, of whom placental tissue was stored for research purposes. For the control group, 46 women were included with a history of ≤1 miscarriage, of whom placental tissue of a healthy singleton pregnancy was stored for research purposes after delivery at the Department of Obstetrics and Gynaecology, LUMC.

For additional experiments, we collected products of conception from eight first trimester miscarriages (GA: 6–10 weeks) and four first trimester elective abortions (GA: 5–10 weeks). The miscarriage material was obtained from women with a history of RM from the Department of Obstetrics and Gynaecology in the LUMC. Elective abortion material was received anonymously from an abortion clinic [36].

The Ethical committee of the LUMC (P11.196, date of approval 18th of June 2012) approved the protocol, and all of the participants gave informed consent for inclusion in the study.

### 4.2. HLA-G Polymorphisms and Haplotypes

Peripheral blood and umbilical cord blood for both groups was processed to genotype *HLA-G* in the mothers and children, respectively. Genomic DNA was isolated to sequence a 699/713-bp fragment covering the 3′UTR of exon 8, starting just before the 14-bp insertion/deletion and ending 591-bp downstream of the insertion/deletion. To sequence the haplotype on each of the two alleles, amplification reactions were performed while using the generic 3′-primer that was tailed with a M13 sequence to cover the 3′UTR region of *HLA-G*. The following polymorphisms were identified: the 14-bp insertion/deletion (rs371194629), +3003C/T (rs1707), +3010C/G (rs1710), +3027A/C (rs17179101), +3035C/T (rs17179108), +3142C/G (rs1063320), +3187A/G (rs9380142), +3196C/G (rs1610696), +3422C/T (rs17875408), +3496A/G (rs1233330), and +3509G/T (rs1611139).

UTR haplotypes were composed based on the combination of SNPs. The conversion of sequencing data to UTR haplotypes was carried out by using specialized HLA interpretation software (version 3.19, SBT Engine, GenDX, Utrecht, The Netherlands). The forward primer (GTGATGGGCTGTTTAAAGTGTCACC), the reverse primer (GACGTTGTAAAACGACGGCCAGTAGGGGAAGAGGTGTAGGGGTCTG), and an M13 universal primer (GACGTTGTAAAACGACGGCCAGT) were ordered from Sigma (St. Louis, MI, USA). The underlining represents the M13 sequence.

### 4.3. Immunohistochemistry

HLA-G and trophoblasts were detected by standard immunohistochemical procedures. After delivery, the placental tissues were dissected and fixed in 4% neutral buffered formaldehyde and embedded in paraffin. Section slides of 6 µm were cut, mounted on Superfrost/Plus glass slides (Thermo Scientific, Waltham, MA, USA), and dried overnight at 37 °C. Sections were deparaffinized in xylene and ethanol. Depending on the primary antibody used, unmasking of the antigens was achieved by enzyme digestion with trypsin or incubation with citrate buffer in a microwave. This was followed by endogenous peroxidase blocking in 3% H_2_O_2_ in methanol for enzymatic staining. All of the incubations were at room temperature and wash steps in between the incubations were performed in PBS. Slides were pre-incubated with PBS/1% BSA to reduce background staining. Excess buffer was removed and slides were incubated with mouse monoclonal primary antibodies overnight at room temperature. Antibodies were diluted in PBS containing 1% BSA.

For enzymatic and immunofluorescence staining, primary antibodies against the free heavy chain of all HLA-G isoforms (MEM-G2; EXBIO Praha, Czech Republic) and against cytokeratin 8 (CAM5.2; Becton Dickinson, Franklin Lakes, NJ, USA) were used. The next day, incubation with secondary antibody (EnVision solution, goat anti-mouse HRP, undiluted; DAKO, Agilent, Santa Clara, CA, USA) for enzymatic staining; Goat-anti-mouse IgG1-AF488 A21121 and Goat-anti-mouse IgG2a-AF546 A21133 for immunofluorescence staining; Thermo Scientific) was performed, and the substrate was visualized with diaminobenzidine (DAB metal Enhanced substrate kit; 34065; Thermo Scientific) for enzymatic staining. Specimens were counterstained with hematoxylin and then mounted in Micromount Mounting Medium (Leica, Nussloch, Germany) for enzymatic staining and ProLong Gold Antifade Mountant with DAPI (P36931; Thermo Scientific) for immunofluorescence staining.

### 4.4. Quantification of Immunohistochemical Stainings

We set out to compare the extent of MEM-G2 and CAM5.2 staining in term placentas between the study groups. All of the slides were scanned by a Pannoramic Midi scanner (version 11, 3DHISTECH, Budapest, Hungary). The entire decidua basalis was quantitatively analyzed using the HistoQuant modus in Quant Center software (version 2.1, 3DHISTECH). This was done by two investigators (JS and HK) independently for 10 placentas to analyze interobserver variability. For each staining, the same thresholds and training scenarios were used for patient and control slides. We corrected for the selected surface area when calculating the percentage positivity of a staining.

For first trimester material, we could not define the decidua. Therefore, we only analyzed the HLA-G positive parts of the slides. Scoring of the slides was performed by two investigators (JS and ME) independently, blinded for the cause of the abortion. Based on the extent of staining, cases were classified according to a semi-quantitative scoring system, i.e., (1) minimal, (2) moderate, or (3) intense staining. Examples of stainings are shown in Figure S1.

### 4.5. RNA Isolation and qPCR

Tissue homogenates from term placentas were processed for mRNA quantification of HLA-G by real-time qPCR. Tissue sections were immersed in ML lysis buffer (Nucleospin miRNA isolation kit from Macherey-Nagel, Düren, Germany) and stored at −20 °C until isolation. RNA was extracted using NucleoSpin columns (Macherey-Nagel) and tested for integrity by gel electrophoresis (Experion, Bio-Rad, Hercules, CA, USA). RNA quantity was determined on a NanoDrop 2000 Spectrophotometer (Thermo Scientific). RNA was combined with oligo DT (Promega; 0.5 µg), dNTP (Promega; 10 mM), and random nucleotide hexamers (0.5 µg; Promega, Fitchburg, WI, USA). This mixture was incubated at 65 °C for five minutes and then put on ice. Complementary DNA synthesis from mRNA was carried out using Superscript III (40 µg/µL RNAseOUT, SuperScriptIII 200 µg/µL, 0.1M DTT; Promega). The reactions were proceeded at 25 °C for five minutes and 50 °C for 60 min. The reactions were terminated by increasing the temperature to 70 °C for 5 min.

PCR assays were carried out using iQ™ SYBR^®^ Green Supermix (Bio-Rad) on a Viia7 Real-time PCR system (Applied Biosystems, Foster City, CA, USA). The PCR program consisted of 10 min at 95 °C, followed by 40 cycles of 15 s at 95 °C and one minute at 60 °C. The levels of mRNA transcripts for HLA-G were normalized to the geometric mean signal of reference genes GAPDH and β-actin. The forward (ACCCACTCCTCCACCTTTGAC) and reverse (TCCACCACCCTGTTGCTGTAG) primer for GAPDH; the forward (ACCACACCTTCTACAATGAG) and reverse (TAGCACAGCCTGGATAGC) primer for beta-actin; the forward (GACAGCGACTCGGCGT) and reverse (GTGTTCCGTGTCTCCTCT) primer for HLA-G were ordered from Sigma.

We also studied miRNA levels in the tissue homogenates. For this, the RNA template was reverse transcribed into cDNA using the miRCURY LNA^TM^ Universal RT miR PCR kit (Exiqon, Vedbaek, Denmark). LNA^TM^ enhanced primer sets were used targeting the following miRNAs of interest: hsa-miR-148a (MIMAT0000243), hsa-miR-148b (MIMAT0000759), hsa-miR-152 (MIMAT0000438), and hsa-miR-365 (MIMAT0000710). Levels of these miRNAs were normalized to the geometric mean signal of previously described reference genes hsa-miR-16 (MIMAT0000069) and hsa-miR-103 (MIMAT0000101) [37,38].

All of the PCR reactions were performed in duplicate. Signals were normalized using the ΔΔCq method. Quantitative PCR measurements were analyzed using QuantStudio Real-Time PCR System Software (Applied Biosystems). To verify the accuracy of amplification, melting curve analyses were performed at the end of each PCR run.

### 4.6. Statistical Analysis

Spearman’s correlation analysis and Bland–Altman plotting were performed for the assessments of validity and reproducibility [17]. Differences between the groups were tested by Mann–Whitney U tests, chi-square tests, or logistic regression analysis. Values of *p* < 0.05 were considered to indicate statistical significance. Association between *HLA-G* SNPs and RM was studied with binary logistic regression. Per *HLA-G* genotype the highest prevalence was defined as the reference group. Alleles with a frequency of <5% were excluded from analysis. For the calculations on the *HLA-G* genotypes, Bonferroni adjustment was used to correct for multiple comparisons. Observed heterozygosity in both groups was computed by the direct counting method. Adherences of genotypic proportions to expectations under Hardy–Weinberg equilibrium were tested separately for each SNP using the PyPop 0.7.0 software (Berkeley, CA, USA) [39]. Statistical analyses were performed using GraphPad Prism version 7.02 for Windows (GraphPad Software, San Diego, CA, USA) and SPSS Statistics 23 (IBM SPSS Software, Armonk, NY, USA).

## Figures and Tables

**Table 1:** Subject characteristics.

Parameters	Pregnancy after RM (*n* = 23)	Uneventful Pregnancy (*n* = 46)	*p*-Value *
Maternal age at time of index pregnancy in years	34 (22–39) ^#^	33 (20–41) ^#^	0.548
Gestational age at time of birth in weeks	39 (37–41) ^#^	39 (37–42) ^#^	0.109
Gravidity at time of index pregnancy	5 (4–9) ^#^	3 (1–7) ^#^	<0.001
Parity at time of index pregnancy	0 (0–2) ^#^	1 (0–5) ^#^	<0.001
Number of previous miscarriages	4 (3–7) ^#^	0 (0–1) ^#^	<0.001

* Mann-Whitney U Test; ^#^ median, min-max.

**Table 2:** The 14-bp insertion/deletion in the 3 prime untranslated region (3′UTR) region of *HLA-G* in the women with a history of recurrent miscarriages (RM) and the control groups.

	RM Women (*n* = 23)		Control Women (*n* = 44) *		OR	95% CI	*p*-Value ^$^
**Genotype Frequency**							
Del/Del	4	17.4%	19	43.2%	0.28	0.08–0.95	0.039
Ins/Del	15	65.2%	16	36.4%	3.28	1.14–9.43	0.026
Ins/Ins	4	17.4%	9	20.5%	0.82	0.22–3.01	0.810
**Phenotype Frequency**							
Ins phenotype ^#^	19	82.6%	25	56.8%	3.61	1.05–12.38	0.039
Del phenotype ^&^	19	82.6%	35	79.6%	1.22	0.33–4.50	0.810
**Allele Frequency**							
Insertion	23	50.0%	34	38.6%	1.59	0.77–3.26	0.205
Deletion	23	50.0%	54	61.4%	0.63	0.31–1.29	0.205

* In two control subjects, the 14bp ins/del could not be defined (4%). ^$^ Chi-square. OR, odds ratio; 95% CI, 95% confidence interval; del, deletion; ins, insertion; ^#^ ins: ins/ins and ins/del; ^&^ del: del/del and ins/del.

**Table 3:** The 14-bp insertion/deletion in the 3′UTR region of *HLA-G* in the offspring of the group with a history of RM and the control group.

	RM Offspring (*n* = 23)		Control Offspring (*n* = 45) *		OR	95% CI	*p*-Value ^$^
**Genotype Frequency**							
Del/Del	8	34.8%	16	34.0%	0.97	1.33–2.77	0.969
Ins/Del	11	47.8%	22	46.8%	0.96	0.35–2.62	0.936
Ins/Ins	4	17.4%	7	14.9%	1.14	0.30–4.39	0.789
**Phenotype Frequency**							
Ins phenotype ^#^	15	65.2%	29	61.7%	1.03	0.36–2.97	0.969
Del phenotype ^&^	19	82.6%	38	84.4%	0.88	0.23–3.36	0.789
**Allele Frequency**							
Insertion	19	43.3%	36	40.0%	1.06	0.51–2.17	0.875
Deletion	27	58.7%	54	60.0%	0.95	0.46–1.95	0.875

* In one control subject the 14bp ins/del could not be defined (2%). ^$^ Chi-square. OR, odds ratio; 95% CI, 95% confidence interval; del, deletion; ins, insertion; ^#^ ins: ins/ins and ins/del; ^&^ del: del/del and ins/del.

**Table 4:** HLA-G protein expression in the placentas of women with a history of RM and controls.

	RM Women (*n* = 23)		Control Women (*n* = 44) *		OR	95% CI	*p*-Value
Low HLA-G protein expression	3	13.0%	23	50.0%	6.67	1.74–25.57	0.006 ^$^
High HLA-G protein expression	20	87.0%	23	50.0%			

^$^ logistic regression. OR, odds ratio; 95% CI, 95% confidence interval.

**Table 5:** HLA-G mRNA expression in the placentas of women with a history of RM and controls.

	RM Women (*n* = 22) *		Control Women (*n* = 32) *		OR	95% CI	*p*-Value
Low HLA-G mRNA expression	3	13.6%	16	50.0%	6.33	1.56–25.71	0.010 ^$^
High HLA-G mRNA expression	19	86.4%	16	50.0%			

* In one RM case (4%) and 12 control subjects (26%) mRNA expression could not be defined. ^$^ logistic regression. OR, odds ratio; 95% CI, 95% confidence interval.

## Supplementary Materials

Supplementary materials can be found at http://www.mdpi.com/1422-0067/20/3/625/s1. Figure S1: Examples of minimal (**A**), moderate (**B**), and intense (**C**) HLA-G staining in first trimester placenta; Figure S2: (**A**) Cytokeratin 8 (CAM5.2, red) and HLA-G (MEM-G2, green) colocalize in the decidua; yellow in merged image indicates overlap of red and green labels. (**B**) CAM5.2 stains all the trophoblasts in the placenta. (**C**) MEM-G2 staining is limited to the extravillous trophoblasts in the decidual part of the placenta; Figure S3: Bland-Altman plot of interobserver measurements of HLA-G staining; Figure S4: HLA-G expression in placentas of healthy first pregnancies compared to subsequent pregnancies; Figure S5: miRNA expression in term placentas of women with a history of RM and controls; Figure S6: MMP2 and MMP9 mRNA expression in term placentas of women with a history of RM and controls. Table S1: Hardy-Weinberg analyses for *HLA-G* 3′UTR genotypes in women with RM and controls; Table S2: Hardy-Weinberg analyses for *HLA-G* 3′UTR genotypes in the RM and control offspring; Table S3: Haplotypes of women in the RM group and the control group; Table S4: *HLA-G* 3′UTR genotypic polymorphisms in women with recurrent miscarriage and uneventful pregnancy; Table S5: Haplotypes of the offspring in the RM group and the control group; Table S6: *HLA-G* 3′UTR genotypic polymorphisms in the offspring of women with recurrent miscarriage and uneventful pregnancy.

## Abbreviations

3′UTR	Three Prime Untranslated Region
DNA	Deoxyribonucleic Acid
EVT	Extravillous Trophoblast
GA	Gestational Age
HLA	Human Leukocyte Antigen
MMP	Matrix Metalloproteinase
NK	Natural Killer
OR	Odds Ratio
PCR	Polymerase Chain Reaction
RM	Recurrent Miscarriage
sHLA	Soluble HLA
SNP	Single Nucleotide Polymorphism
