# Supplementary material for: Increased HLA-G Expression in Term Placenta of Women with a History of Recurrent Miscarriage Despite Their Genetic Predisposition to Decreased HLA-G Levels
Source: Int J Mol Sci. 2019 Feb 1;20(3):625. doi: 10.3390/ijms20030625 (PMC6387365; doi:10.3390/ijms20030625)
Supplement: Supplementary file 1 [file ijms-20-00625-s001.zip › ijms-421619-supplementary-proofreading/HLA-G in RM_Supplementary -figures.pdf]

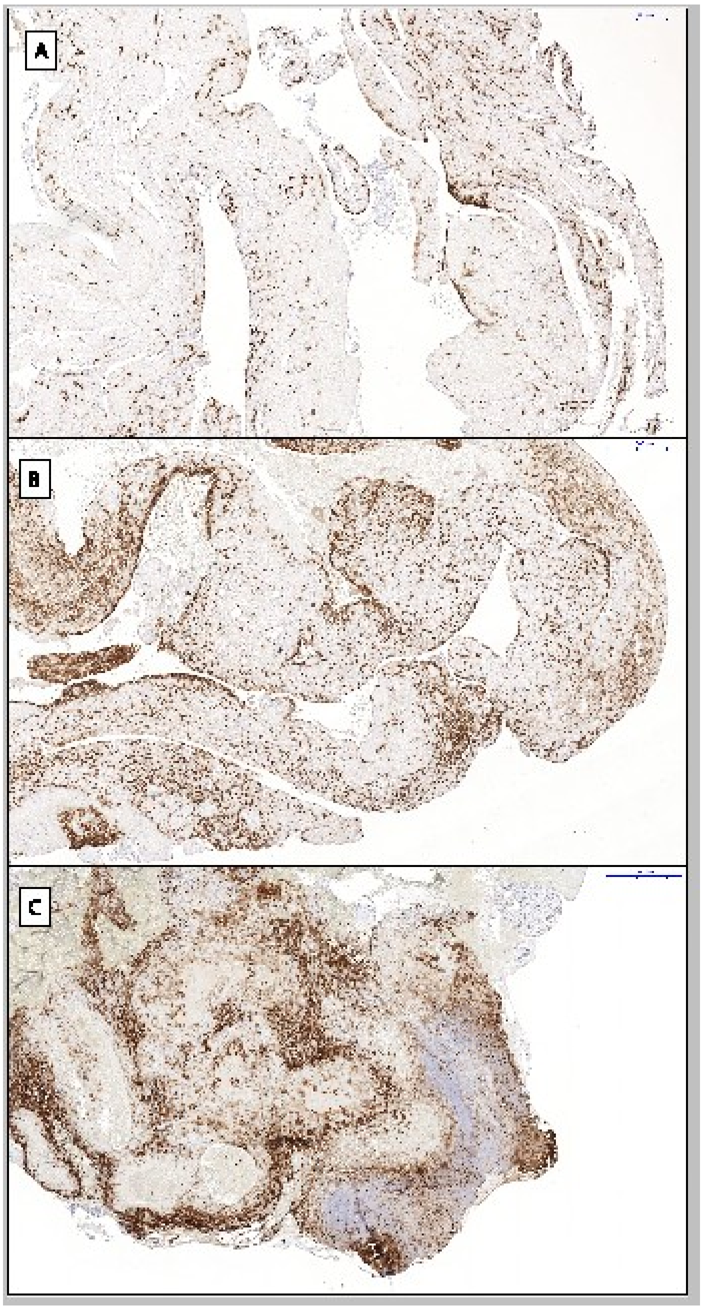

Figure S1. Examples of minimal (**A**), moderate (**B**), and intense (**C**) HLA-G staining in first trimester placenta.

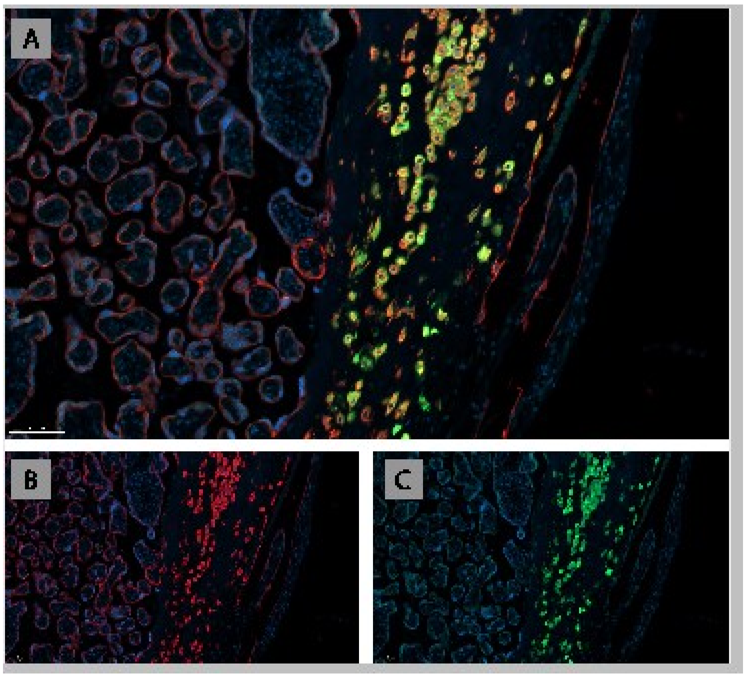

Figure S2. (**A**) Cytokeratin 8 (CAM5.2, red) and HLA-G (MEM-G2, green) colocalize in the decidua; yellow in merged image indicates overlap of red and green labels. (**B**) CAM5.2 stains all the trophoblasts in the placenta. (**C**) MEM-G2 staining is limited to the extravillous trophoblasts in the decidual part of the placenta.

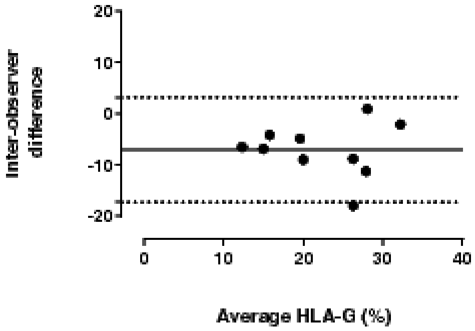

Figure S3. Bland-Altman plot of interobserver measurements of HLA-G staining. Ninety percent of the values ranged within a mean ± 2 SD deviations, indicating acceptable reproducibility.

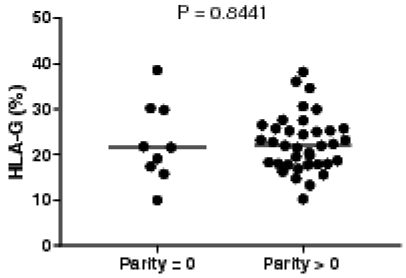

Figure S4. HLA-G expression in placentas of healthy first pregnancies compared to subsequent pregnancies. Previous pregnancies did not influence placental HLA-G expression in the control group with uncomplicated pregnancies.

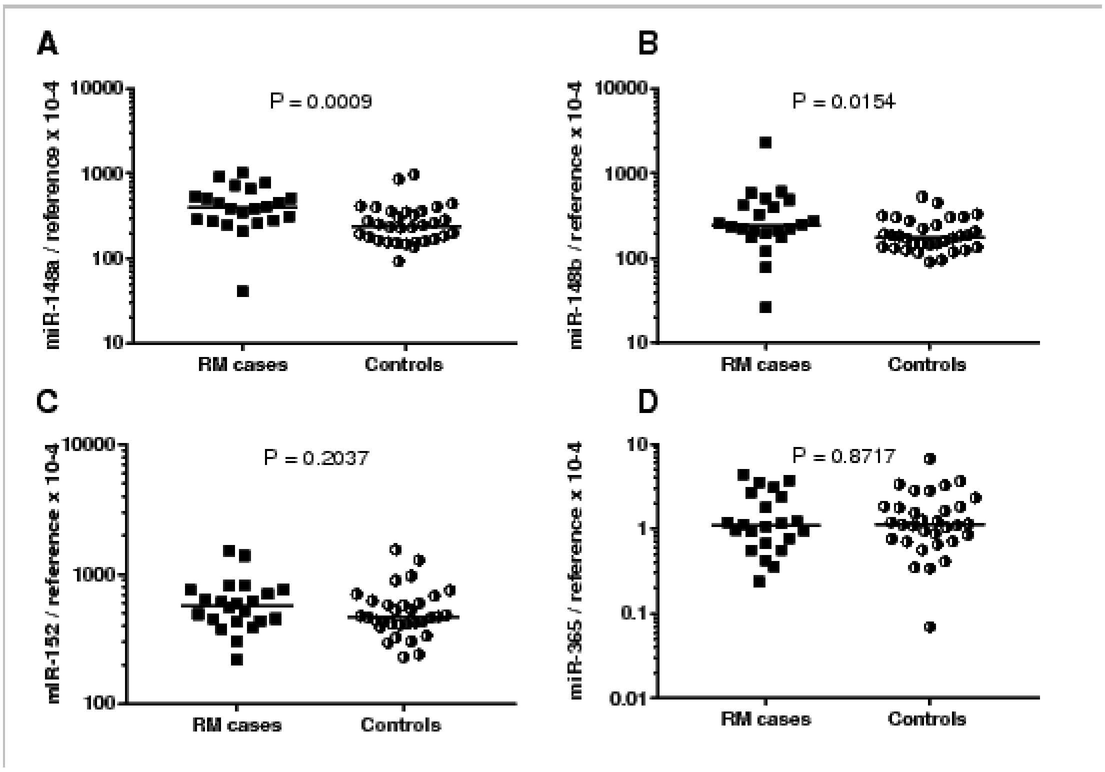

Figure S5. miRNA expression in term placentas of women with a history of RM and controls. (**A**,**B**) miR-148a and miR-148b expression was elevated in placenta of women with a history of RM compared to controls. No difference in miRNA expression was seen for (**C**) miR-152 and (**D**) miR-365 (statistics: Mann-Whitney test).

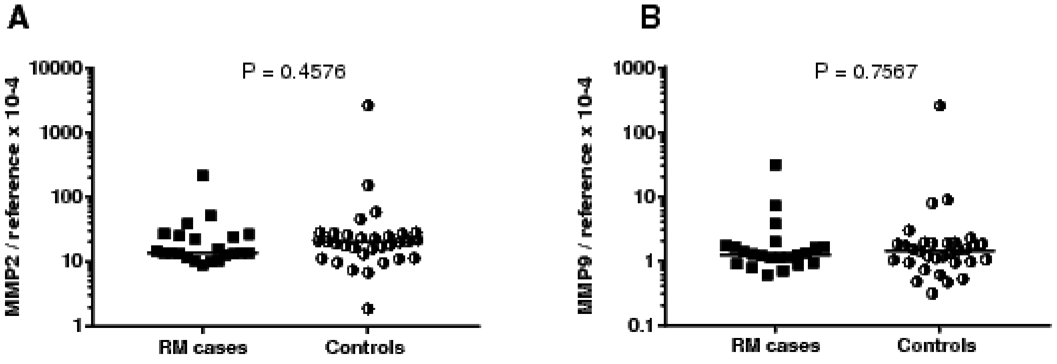

Figure S6. MMP2 and MMP9 mRNA expression in term placentas of women with a history of RM and controls. (**A**) MMP2 mRNA expression was similar between women with a history of RM and controls. (**B**) MMP9 mRNA expression was similar between women with a history of RM and controls (statistics: Mann-Whitney test).
