# Supplementary material for: Increased HLA-G Expression in Term Placenta of Women with a History of Recurrent Miscarriage Despite Their Genetic Predisposition to Decreased HLA-G Levels
Source: Int J Mol Sci. 2019 Feb 1;20(3):625. doi: 10.3390/ijms20030625 (PMC6387365; doi:10.3390/ijms20030625)
Supplement: Supplementary file 1 [file ijms-20-00625-s001.zip › ijms-421619-supplementary-proofreading/HLA-G in RM_Supplementary Table_S1.pdf]

Supplementary Table S1. Hardy-Weinberg analyses for HLA-G 3'UTR genotypes in women with RM and controls.

SNP	*Recurrent miscarriage (n=23)*			*Controls (n=46)*		
	common *P*	homozygotes *P*	heterozygote *P*	common *P*	homozygotes *P*	heterozygotes *P*
14-bp	0.1444	0.3020	0.3020	0.1220	0.3119	0.2870
+3003	#	0.9370	&	0.5888	0.7548	0.6054
+3010	0.7171	0.7637	0.7526	0.1379	0.2975	0.2953
+3027	#	0.9653	&	0.8602	0.9474	0.8626
+3035	0.6481	0.7965	0.6619	0.7823	0.9045	0.7874
+3142	0.7171	0.7637	0.7526	0.2322	0.3997	0.3987
+3187	0.5487	0.6508	0.5836	0.3077	0.3077	0.2440
+3196	0.4238	0.5347	0.4688	0.2315	0.3908	0.2679
+3422	0.9250	0.9520	0.9292	0.5149	0.7170	0.5321
+3496	#	0.8996	&	0.5888	0.7548	0.6054
+3509	0.5855	0.6588	0.6280	0.2938	0.4428	0.3332

All Hardy-Weinberg analyses. *P*, p value. # Too many parameters for chi-square test. & Too few cases to calculate p-value.
