# Supplementary material for: Increased HLA-G Expression in Term Placenta of Women with a History of Recurrent Miscarriage Despite Their Genetic Predisposition to Decreased HLA-G Levels
Source: Int J Mol Sci. 2019 Feb 1;20(3):625. doi: 10.3390/ijms20030625 (PMC6387365; doi:10.3390/ijms20030625)
Supplement: Supplementary file 1 [file ijms-20-00625-s001.zip › ijms-421619-supplementary-proofreading/HLA-G in RM_Supplementary Table_S2.pdf]

Supplementary Table S2. Hardy-Weinberg analyses for HLA-G 3'UTR genotypes in the RM and control offspring.

SNP	*Recurrent miscarriage (n=23)*			*Controls (n=46)*		
	common *P*	homozygotes *P*	heterozygote *P*	common *P*	homozygotes *P*	heterozygotes *P*
14-bp	0.9577	0.9647	0.9637	0.9011	0.9341	0.9314
+3003	0.9471	0.9644	0.9504	0.8976	0.9458	0.9013
+3010	0.2058	0.2920	0.2883	0.3181	0.4854	0.4820
+3027	#	0.9653	&	#	0.9877	&
+3035	0.7224	0.8528	0.7319	0.7009	0.8533	0.7094
+3142	0.2921	0.4575	0.4567	0.8841	0.9179	0.9179
+3187	0.8147	0.8691	0.8275	0.7358	0.7894	0.7617
+3196	0.5487	0.6508	0.5836	0.7862	0.8389	0.8038
+3422	0.4882	0.6574	0.5128	0.9110	0.9424	0.9161
+3496	0.9471	0.9644	0.9504	0.8976	0.9458	0.9013
+3509	0.5487	0.6508	0.5836	#	0.8150	0.7861

All Hardy-Weinberg analyses. *P*, p value. # Too many parameters for chi-square test. & Too few cases to calculate p-value.
