# Supplementary material for: Increased HLA-G Expression in Term Placenta of Women with a History of Recurrent Miscarriage Despite Their Genetic Predisposition to Decreased HLA-G Levels
Source: Int J Mol Sci. 2019 Feb 1;20(3):625. doi: 10.3390/ijms20030625 (PMC6387365; doi:10.3390/ijms20030625)
Supplement: Supplementary file 1 [file ijms-20-00625-s001.zip › ijms-421619-supplementary-proofreading/HLA-G in RM_Supplementary Table_S3.pdf]

Supplementary Table S3. Haplotypes of women in the RM group and the control group.

	RM women 2n=46		*Controls 2n=88**		**OR**	**95% C.I.**		**P**
						**Lower**	**Upper**	
UTR-1	13	28.3%	28	31.8%	0.90	0.413	1.965	0.792
UTR-2	16	34.8%	23	26.1%	1.60	0.742	3.451	0.231
UTR-3	5	10.9%	8	9.1%	1.28	0.394	4.159	0.681
UTR-4	5	10.9%	14	15.9%	0.68	0.229	2.019	0.487
UTR-5	4	8.7%	4	4.5%	2.10	0.499	8.789	0.312
UTR-7	3	6.5%	7	8.0%	0.85	0.209	3.440	0.817
UTR-8	0	0.0%	0	0.0%	x	x	x	x
UTR-18	0	0.0%	3	3.4%	0.00	0.000		0.999
UTR-N	0	0.0%	1	1.1%	0.00	0.000		1.000
	46	100%	88	100%				

All univariate logistic regression analysis. *P*, *p* value; *OR*, odds ratio; *95% CI*, 95% confidence interval; n.a, not applicable. *In 2 control subjects the UTR haplotype could not be defined (4%). The 3'UTR haplotype nomenclature is consistent with publication by Castelli et al.
