# Supplementary material for: Increased HLA-G Expression in Term Placenta of Women with a History of Recurrent Miscarriage Despite Their Genetic Predisposition to Decreased HLA-G Levels
Source: Int J Mol Sci. 2019 Feb 1;20(3):625. doi: 10.3390/ijms20030625 (PMC6387365; doi:10.3390/ijms20030625)
Supplement: Supplementary file 1 [file ijms-20-00625-s001.zip › ijms-421619-supplementary-proofreading/HLA-G in RM_Supplementary Table_S4.pdf]

Supplementary Table S4. HLA-G 3'UTR genotypic polymorphisms in women with recurrent miscarriage and uneventful pregnancy.

		RM women (n=23)		*Controls (n=44)*		*OR*	*95% CI*	*P*	*Pc*
**3003**	**CC**	0	0.0%	2	4.5%	n.c.			
	**CT**	4	17.4%	10	22.7%	0.67	0.19-2.45	0.549	1.000
	**TT**	19	82.6%	32	72.7%	ref.			
**3010**	**CC**	8	34.8%	12	27.3%	0.94	0.30-3.01	0.923	1.000
	**CG**	12	52.2%	17	38.6%	ref.			
	**GG**	3	13.0%	15	34.1%	0.28	0.07-1.20	0.087	1.000
**3027**	**AA**	0	0.0%	0	0.0%	n.c.			
	**AC**	3	13.0%	6	13.6%	0.95	0.22-4.21	0.946	1.000
	**CC**	20	87.0%	38	86.4%	ref.			
**3035**	**CC**	16	69.6%	36	81.8%	ref.			
	**CT**	7	30.4%	8	18.2%	1.97	0.61-6.36	0.258	1.000
	**TT**	0	0.0%	0	0.0%	n.c.			
**3142**	**CC**	3	13.0%	14	31.8%	0.32	0.08-1.36	0.124	1.000
	**CG**	12	52.2%	18	40.9%	ref.			
	**GG**	8	34.8%	12	27.3%	1.00	0.32-3.17	1.000	1.000
**3187**	**AA**	11	47.8%	23	52.3%	ref.			
	**AG**	11	47.8%	14	31.8%	1.64	0.57-4.48	0.362	1.000
	**GG**	1	4.3%	7	15.9%	0.30	0.03-2.74	0.285	1.000
**3196**	**CC**	10	43.5%	27	61.4%	ref.			
	**CG**	12	52.2%	12	27.3%	2.70	0.92-7.95	0.071	1.000
	**GG**	1	4.3%	5	11.4%	n.c.			
**3422**	**CC**	15	65.2%	33	75.0%	ref.			
	**CT**	7	30.4%	9	20.5%	1.71	0.54-5.46	0.365	1.000
	**TT**	1	4.3%	2	4.5%	n.c.			
**3496**	**AA**	0	0.0%	2	4.5%	n.c.			
	**AG**	5	21.7%	10	22.7%	0.89	0.26-3.01	0.850	1.000
	**GG**	18	78.3%	32	72.7%	ref.			
**3509**	**GG**	9	39.1%	26	59.1%	ref.			
	**GT**	12	52.2%	13	29.5%	2.67	0.90-7.94	0.078	1.000
	**TT**	2	8.7%	5	11.4%	1.16	0.19-7.04	0.875	1.000

Data are all n (%). All univariate logistic regression analysis. Per HLA-G genotype the highest prevalence was defined as the reference group. If percentages in a group were below 5%, no calculations were performed. *P*, *p* value; *Pc*, *p* value corrected for multiple comparisons; *OR*, odds ratio; *95% CI*, 95% confidence interval; n.c, not calculated; ref, reference group.
