# Supplementary material for: Increased HLA-G Expression in Term Placenta of Women with a History of Recurrent Miscarriage Despite Their Genetic Predisposition to Decreased HLA-G Levels
Source: Int J Mol Sci. 2019 Feb 1;20(3):625. doi: 10.3390/ijms20030625 (PMC6387365; doi:10.3390/ijms20030625)
Supplement: Supplementary file 1 [file ijms-20-00625-s001.zip › ijms-421619-supplementary-proofreading/HLA-G in RM_Supplementary Table_S5.pdf]

**Supplementary Table S5. Haplotypes of the offspring in the RM group and the control group.**

| | *RM offspring 2n=46* | | *Controls 2n=90** | | OR | 95% C.I. | | P |
|---|---|---|---|---|---|---|---|---|
| | | | | | | Lower | Upper | |
| UTR-1 | 11 | 23.9% | 28 | 31.1% | 0.70 | 0.309 | 1.566 | 0.381 |
| UTR-2 | 13 | 28.3% | 26 | 28.9% | 0.97 | 0.441 | 2.131 | 0.939 |
| UTR-3 | 6 | 13.0% | 11 | 12.2% | 1.08 | 0.371 | 3.125 | 0.891 |
| UTR-4 | 10 | 21.7% | 12 | 13.3% | 1.81 | 0.714 | 4.565 | 0.212 |
| UTR-5 | 3 | 6.5% | 7 | 7.8% | 0.83 | 0.204 | 3.360 | 0.791 |
| UTR-7 | 3 | 6.5% | 3 | 3.3% | 2.02 | 0.392 | 10.446 | 0.400 |
| UTR-8 | 0 | 0.0% | 1 | 1.1% | 0.00 | 0.000 | | 1.000 |
| UTR-18 | 0 | 0.0% | 2 | 2.2% | 0.00 | 0.000 | | 0.999 |
| UTR-N | 0 | 0.0% | 0 | 0.0% | x | x | x | x |
| | 46 | 100% | 90 | 100% | | | | |

All univariate logistic regression analysis. *P*, *p* value; *OR*, odds ratio; *95% CI*, 95% confidence interval; n.a, not applicable. *In 1 control subject the UTR haplotype could not be defined (2%). The 3'UTR haplotype nomenclature is consistent with publication by Castelli et al.
