# Supplementary material for: Increased HLA-G Expression in Term Placenta of Women with a History of Recurrent Miscarriage Despite Their Genetic Predisposition to Decreased HLA-G Levels
Source: Int J Mol Sci. 2019 Feb 1;20(3):625. doi: 10.3390/ijms20030625 (PMC6387365; doi:10.3390/ijms20030625)
Supplement: Supplementary file 1 [file ijms-20-00625-s001.zip › ijms-421619-supplementary-proofreading/HLA-G in RM_Supplementary Table_S6.pdf]

Supplementary Table S6. HLA-G 3'UTR genotypic polymorphisms in the offspring of women with recurrent miscarriage and uneventful pregnancy.

		RM offspring (n=23)		*Controls (n=45)*		*OR*	*95% CI*	*P*	*Pc*
**3003**	**CC**	1	4.3%	1	2.2%	n.c.			
	**CT**	8	34.8%	10	22.2%	1.94	0.63-5.95	0.245	1.000
	**TT**	14	60.9%	34	75.6%	ref.			
**3010**	**CC**	5	21.7%	11	24.4%	0.58	0.16-2.02	0.389	1.000
	**CG**	15	65.2%	19	42.2%	ref.			
	**GG**	3	13.0%	15	33.3%	0.25	0.06-1.04	0.057	0.969
**3027**	**AA**	0	0.0%	0	0.0%	n.c.			
	**AC**	3	13.0%	3	6.7%	2.10	0.39-11.34	0.389	1.000
	**CC**	20	87.0%	42	93.3%	ref.			
**3035**	**CC**	17	73.9%	35	77.8%	ref.			
	**CT**	6	26.1%	10	22.2%	1.24	0.38-3.97	0.722	1.000
	**TT**	0	0.0%	0	0.0%	n.c.			
**3142**	**CC**	5	21.7%	11	24.4%	0.71	0.20-2.50	0.598	1.000
	**CG**	14	60.9%	22	48.9%	ref.			
	**GG**	4	17.4%	12	26.7%	0.52	0.14-1.95	0.335	1.000
**3187**	**AA**	13	56.5%	20	44.4%	ref.			
	**AG**	9	39.1%	21	46.7%	0.66	0.23-1.88	0.436	1.000
	**GG**	1	4.3%	4	8.9%	n.c.			
**3196**	**CC**	11	47.8%	24	53.3%	ref.			
	**CG**	11	47.8%	17	37.8%	1.41	0.50-4.00	0.516	1.000
	**GG**	1	4.3%	4	8.9%	n.c.			
**3422**	**CC**	14	60.9%	29	64.4%	ref.			
	**CT**	9	39.1%	14	31.1%	1.33	0.47-3.82	0.594	1.000
	**TT**	0	0.0%	2	4.4%	n.c.			
**3496**	**AA**	1	4.3%	1	2.2%	n.c.			
	**AG**	8	34.8%	10	22.2%	1.94	0.63-5.95	0.245	1.000
	**GG**	14	60.9%	34	75.6%	ref.			
**3509**	**GG**	11	47.8%	23	51.1%	ref.			
	**GT**	11	47.8%	17	37.8%	1.35	0.48-3.85	0.571	1.000
	**TT**	1	4.3%	4	8.9%	0.52	0.05-5.25	0.581	1.000
	**CT**	0	0.0%	1	2.2%	n.c.			

Data are all n (%). All univariate logistic regression analysis. Per HLA-G genotype the highest prevalence was defined as the reference group. If percentages in a group were below 5%, no calculations were performed. *P*, *p* value; *Pc*, *p* value corrected for multiple comparisons; *OR*, odds ratio; *95% CI*, 95% confidence interval; n.c, not calculated; ref, reference group.
